# NEIGHBORHOOD ADVERSITY AND COGNITIVE HEALTH: THE MODIFYING ROLE OF SELF-PERCEPTIONS OF AGING

**DOI:** 10.1093/geroni/igac059.1606

**Published:** 2022-12-20

**Authors:** Eunyoung Choi, Elizabeth Zelinski, Jennifer Ailshire, Yuri Jang

**Affiliations:** School of Global Public Health, New York University, Los Angeles, California, United States; University of Southern California, Los Angeles, California, United States; University of Southern California, Los Angeles, California, United States; University of Southern California, Los Angeles, California, United States

## Abstract

Research has documented the increased risk of cognitive impairment among older adults living in socioeconomically disadvantaged neighborhoods. Much less is known about the factors that moderate this risk. We conceptualized self-perception of aging (SPA) as a potential moderator because it reflects core beliefs about the self at older ages but is also closely linked to late-life health. Guided by the diathesis-stress model that postulates the interactive roles of cognitive styles and stressors in shaping health outcomes, we hypothesized that more positive SPA would buffer the effects of neighborhood adversity on cognitive function. Using data from the Health and Retirement Study (2008–2016), the analytic sample consisted of adults aged 54 and older (N=5,902). Cognitive function was assessed by the Telephone Interview for Cognitive Status. The neighborhood indicators included 1) poverty rates at the census tract level, 2) perceived neighborhood social cohesion, and 3) perceived neighborhood disorder. Three-level growth curve models were separately estimated for each neighborhood indicator’s effect as well as its interaction with SPA on the 8-year cognitive function trajectories. Findings showed that higher poverty rates, more disorder, and less cohesion were associated with lower initial levels of cognitive function but slower rates of cognitive decline. SPA partially moderated the linkage between neighborhood adversity and the level of cognitive function. More positive SPA was associated with reduced negative effects of living in neighborhoods with higher poverty rates and more physical disorder. These findings highlight the intersection of an individual-level psychological factor and a contextual-level factor in shaping late-life cognition.

